# Screening epitopes on systemic lupus erythematosus autoantigens with a peptide array

**DOI:** 10.18632/oncotarget.20994

**Published:** 2017-09-18

**Authors:** Lin Wang, Chenjun Hao, Yongqiu Deng, Yanbo Liu, Shiliang Hu, Yangang Peng, Manna He, Jinhu Fu, Ming Liu, Jia Chen, Xiaoming Chen

**Affiliations:** ^1^ Department of Rheumatology, Shaoyang Central Hospital, 422000 Shaoyang, China; ^2^ Obstetrics and gynecology, Guangzhou Panyu Hexian Memorial Hospital, 511400 Guangzhou, China

**Keywords:** systemic lupus erythematosus, epitope, peptide array, autoantibody

## Abstract

Systemic lupus erythematosus (SLE) is a common autoimmune disease. Many autoantibodies are closely associated with SLE. However, the specific epitopes recognized and bound by these autoantibodies are still unclear. This study screened the binding epitopes of SLE-related autoantibodies using a high-throughput screening method. Epitope prediction on 12 SLE-related autoantigens was performed using the Immune Epitope Database and Analysis Resource (IEDB) software. The predicted epitopes were synthesized into peptides and developed into a peptide array. Serum IgG from 50 SLE patients and 25 healthy controls was detected using the peptide array. The results were then validated using an enzyme-linked immunosorbent assay (ELISA). The diagnostic efficiency of each epitope was analyzed using a ROC curve. Seventy-three potential epitopes were screened for using the IEDB software after the epitopes on the 12 SLE-related autoantigens were analyzed. Peptide array screening revealed that the levels of the autoantibodies recognized and bound by 4 peptide antigens were significantly upregulated in the serum of SLE patients (*P* < 0.05). The ELISA results showed that the 4 antigens with significantly increased serum autoantibodies levels in SLE patients were acidic ribosomal phosphoprotein (P0)-4, acidic ribosomal phosphoprotein (P0)-11, DNA topoisomerase 1 (full length)-1, and U1-SnRNP 68/70 KDa-1 (*P* < 0.05), and the areas under the ROC curve for diagnosing SLE on the basis of these peptides were 0.91, 0.90, 0.93, and 0.91, respectively. Many autoantibodies specifically expressed in the serum of patients with SLE can be detected by specific peptide fragments and may be used as markers in clinical auxiliary diagnoses.

## INTRODUCTION

Systemic lupus erythematosus (SLE), a diffuse connective tissue disease, is predominantly characterized by immunological inflammation [1, 2]. SLE cases are widely distributed throughout the world, but regional differences in SLE are evident. The incidence of SLE is approximately 50/100,000 in the United States, 15/100,000 in England, and 3.2/100,000 in India, while the incidence in China is 70/100,000 [3–5]. SLE is an autoimmune disease that involves multiple systems, organs, and autoantibodies. SLE can cause serious damage to various systems and organs, such as the skin, joints, serosa, heart, kidneys, central nervous system, and blood system, which seriously endangers the health of SLE sufferers. However, the etiology and pathogenesis of SLE are currently unclear, and both genetic factors and the environment are believed to have some impact on its development. The main pathological manifestation of SLE is the production of numerous autoantibodies directed against proteins within the body, including the anti-nuclear antibody (ANA), anti-double-stranded DNA (dsDNA) antibody, anti-Sm antibody, anti-nucleosome antibody, anti-U1RNP antibody (anti-nRNP antibody), anti-ribosomal P antibody (anti-rRNP antibody), and anti-SSA antibody. ANA is relatively specific for this disease; the positive detection rate for ANA is as high as 89–97%, and it is the most commonly detected autoantibody in SLE patients [6–8].

Since the specificity of ANA for a diagnosis of SLE is only 10–40%, although its sensitivity is as high as 97%-100%, the diagnosis of SLE cannot be completely ruled out when ANA tests are negative. Therefore, clinical conditions should also be taken into account when diagnosing SLE. The anti-dsDNA antibody has a high specificity for the diagnosis of SLE. It is closely related to the symptoms of SLE, especially lupus nephritis, and its titer fluctuates with the activity of the disease. However, in some patients with a severe condition, a high titer of anti-dsDNA antibodies cannot be detected due to excessive free DNA antigens in the serum, which combine with anti-dsDNA antibodies. Approximately 40–75% SLE patients are positive for anti-dsDNA antibodies [9, 10]. The specificity of the anti-Sm antibody for SLE is as high as 98%, but the sensitivity is only 20%-30%. However, enzyme-linked immunosorbent assay (ELISA) detection can increase the sensitivity by 10% without affecting the specificity [11–14]. The titer of the anti-Sm antibody may be related to the activity of SLE; therefore, an elevated titer may indicate SLE recurrence. Anti-Sm antibodies are very helpful in diagnosing early or atypical SLE or in making a retrospective diagnosis of SLE after treatment. The anti-nucleosome antibody is another specific antibody for SLE; the positive detection rate of this antibody is as high as 82–86%. The anti-nRNP antibody has a significant impact on the diagnosis of SLE, and the positive detection rate is 45–60%. Anti-Sm antibodies usually coexist with anti-nRNP antibodies [15]. Anti-rRNP antibodies are mainly present in patients with SLE and are positively correlated with their psychiatric symptoms. The positive detection rate of anti-rRNP antibodies in SLE patients is approximately 20–30%. Anti-SSA antibodies are associated with a variety of autoimmune diseases and are most commonly found in patients with Sjogren’s syndrome (75%), but they are also observed in association with SLE (30–40%), primary biliary cirrhosis (20%), and occasionally chronic active hepatitis. Anti-SSA antibody tests are positive in 100% of neonatal patients with lupus erythematosus. This antibody can be passed to the fetus through the placenta and cause inflammatory responses and neonatal congenital heart block [16, 17]. However, all these current detection methods have limitations, such as low positive detection rates and low sensitivities. Additionally, these antibodies can only be detected using an ELISA, which is not only inefficient but is also costly and is becoming a major obstacle to the early diagnosis of SLE. Therefore, the identification of a simple, fast, and low-cost SLE diagnostic method with good specificity and high sensitivity has become an urgent problem. Scholars have attempted to use protein arrays to detect autoantibodies in patients for diagnosing SLE [18–20]. However, due to the difficulties in obtaining complete and pure autoantigen samples, it is necessary to find effective alternatives.

A peptide array is a type of biochip that has been rapidly developed in recent years. Peptide arrays integrate a variety of active peptides on a very small surface area. Based on trace physiological or biological samples, they can be used to detect and investigate interactions between different biomolecules and functional gene expression [21]. Compared with traditional methods, peptide arrays are simple and fast, with a high throughput, high accuracy, and low cost. They can be easily carried out and are quick and accurate for large-scale screening. Zhong et al. collected patients’ serum to screen for lung cancer indicators using peptide arrays, and the diagnostic accuracy was 93.1% [22]. Later, Chapman et al. improved on the use of relevant indicators using a peptide array to examine the plasma of lung cancer patients, and the diagnostic accuracy reached 92% [23]. Peptide arrays for patients with head and neck cancer have also been found to possess potential applications in the detection of p53 autoantibodies [24]. Peptide arrays have also been used in the study of protein amino acid methylation [25]. However, the use of peptide array techniques in the early diagnosis of autoimmune diseases, especially SLE, or in treatments to block related bare sites has rarely been reported.

In this study, we examined the serum of SLE patients and normal healthy controls using a peptide array and validated the significantly differentially expressed peptides using an ELISA. Our goal was to screen for potential biomarkers for SLE and to explore their significance in clinical applications.

## RESULTS

### General data

The general data for the SLE and control groups are summarized in Table 1. The groups did not show significant differences in their male/female ratio or age (*P* > 0.05).

**Table 1:** Information for patients with active SLE in the peptide array screening group

	SLE patients	Healthy controls
Male/female ratio	1:4	1:4
Mean age	30.7 ± 4.9	26.5 ± 2.1
Proteinuria (g/d)	0.25 ± 0.08*	0.13 ± 0.02
Leukocytes (cells/L)	(4.72 ± 0.79) × 10^9*^	(6.02 ± 0.32) × 10^9^
Medication within 2 months before diagnosis	None	/
Disease duration (months)^1^	3 ± 1.2	/
SLEDAI^2^	4.2 ± 1.2	/

^1^The time span from the appearance of clinical manifestation to doctor’s office visit; ^2^systemic lupus erythematosus disease activity index. *P < 0.05 vs. the control.

### IEDB prediction outcomes

A total of 73 potential antigen peptide indicators were obtained from the 12 predicted antigens using Immune Epitope Database and Analysis Resource (IEDB) software. The detailed information about the indicators is summarized in Supplementary Table 1.

### Peptide array detection outcomes

**Figure 1:**
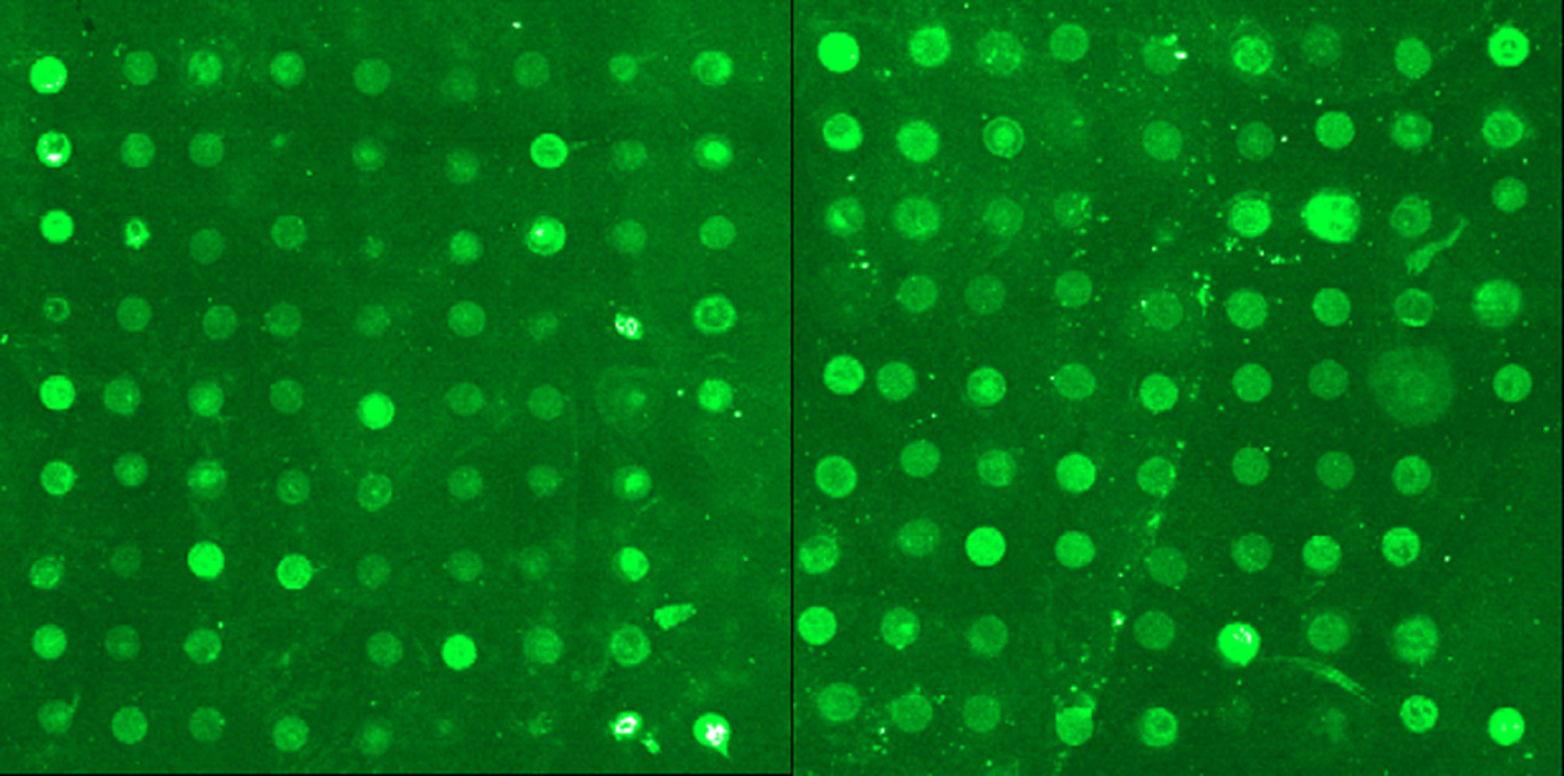
Representative detection results from SLE peptide arrays

A discovery study was carried out in which serum samples from 50 SLE patients and matched healthy controls were hybridized to antibody-coated glass slide arrays that measured the levels of 73 human peptides. Representative detection results are shown in Figure 1.

**Figure 2:**
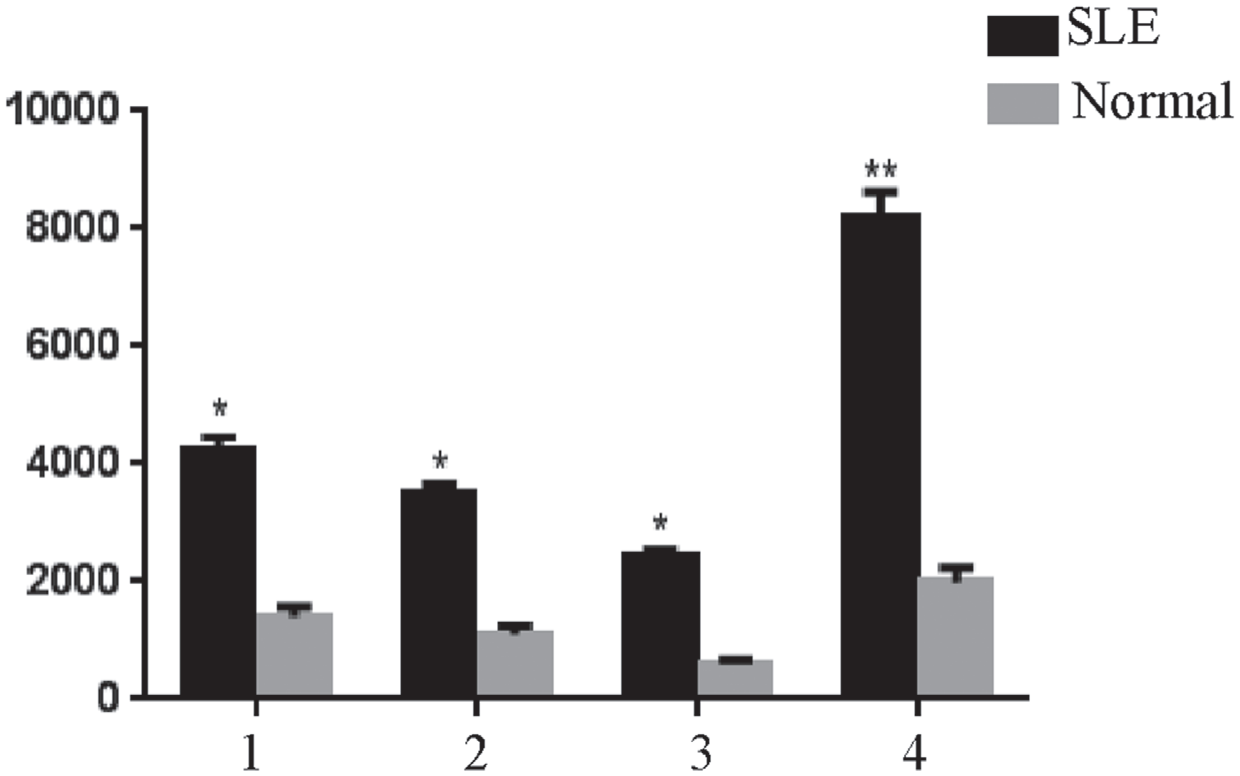
Significantly different expression levels of peptides in serum samples of the normal controls and SLE patients detected using peptide arrays, *P < 0.05, **P < 0.01 vs. the control 1, (P0)-4. 2, (P0)-11. 3, DNA topoisomerase 1-1. 4, U1-SnRNP 68/70 KDa-1.

Gene Pro 6.0 software was used to read the signals and analyze the results. The expression levels of 4 peptides, acidic ribosomal phosphoprotein (P0)-4, acidic ribosomal phosphoprotein (P0)-11, DNA topoisomerase 1 (full length)-1, and U1-SnRNP 68/70 KDa-1, were significantly different between the SLE patients and normal controls (Figure 2, Table 2).

**Table 2:** Data from *t* tests of the significantly differentially expressed peptides detected by SLE diagnostic peptide arrays

Peptide		Levene’s test for equal of variances		*t*-test for equality of means						
		F	Sig.	*t*	df	Sig. (2-tailed)	Mean difference	Std. error difference	95% CI	
									Lower	Upper
(P0)-4	Equal variances assumed	5.43	0.02	2.00	65	0.05	1596.82	800.10	–1.089	3194.72
	Equal variances not assumed			2.02	36.55	0.05	1596.82	789.26	–3.03	3196.66
(P0)-11	Equal variances assumed	1.99	0.16	2.37	65	0.02	664.48	280.86	103.57	1225.40
	Equal variances not assumed			2.39	49.96	0.02	664.48	278.43	105.24	1223.73
DNA topoisomerase 1 (full length)-1	Equal variances assumed	.055	0.82	2.44	65	0.02	794.68	325.89	143.84	1445.51
	Equal variances not assumed			2.43	60.99	.018	794.68	327.00	140.78	1448.50
U1-SnRNP68/70KDa-5	Equal variances assumed	8.013	0.01	3.34	65	.001	1524.16	456.98	611.51	2436.82
	Equal variances not assumed			3.37	44.58	.002	1524.16	452.16	613.23	2435.10

### ELISA outcomes

**Figure 3:**
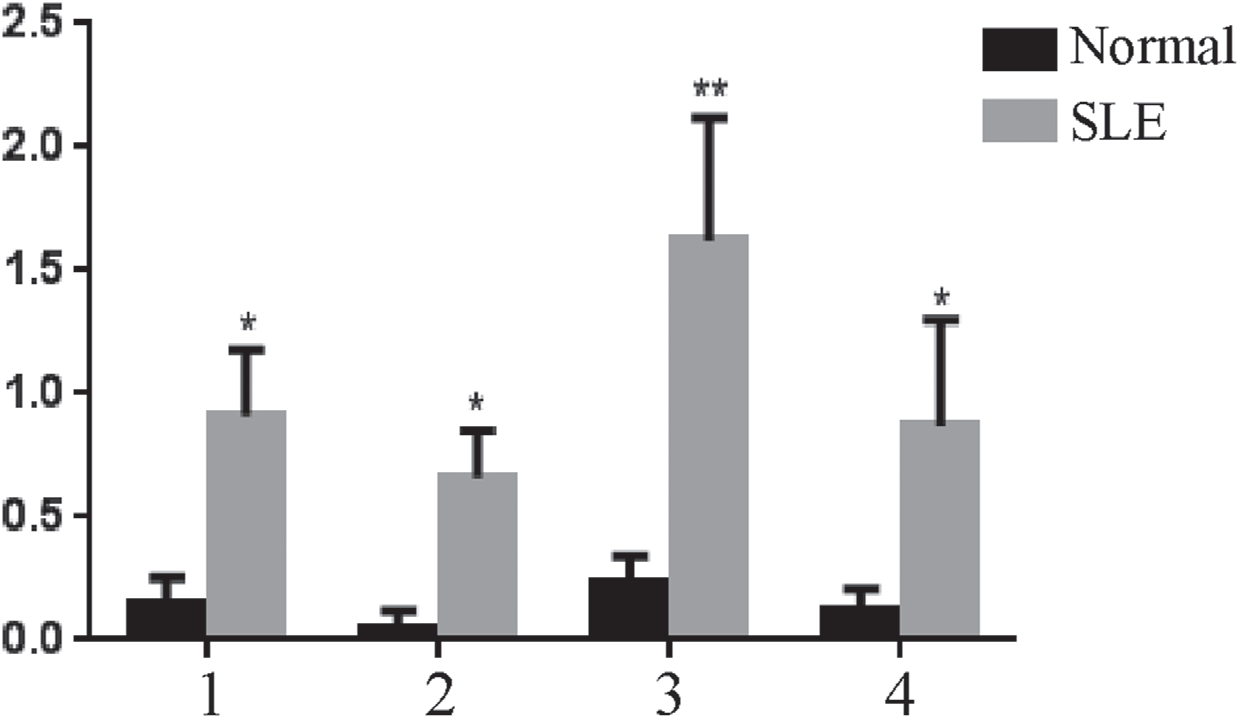
ELISA-based validation for significantly expressed peptides in serum samples, *P < 0.05, **P < 0.01 vs. the control 1: PO-4; 2: PO-11; 3: DNA topoisomerase 1 (full length)-1; 4: U1-SnRNP 68/70 KDa-1.

On the basis of these peptide screens, acidic ribosomal phosphoprotein (P0)-4, acidic ribosomal phosphoprotein (P0)-11, DNA topoisomerase 1 (full length)-1 and U1-SnRNP 68/70 KDa-1 peptides were selected for ELISA-based validation in an independent cohort of 40 SLE patients. The validation results are shown in Figure 3.

### ROC curve plotting and analysis

**Figure 4:**
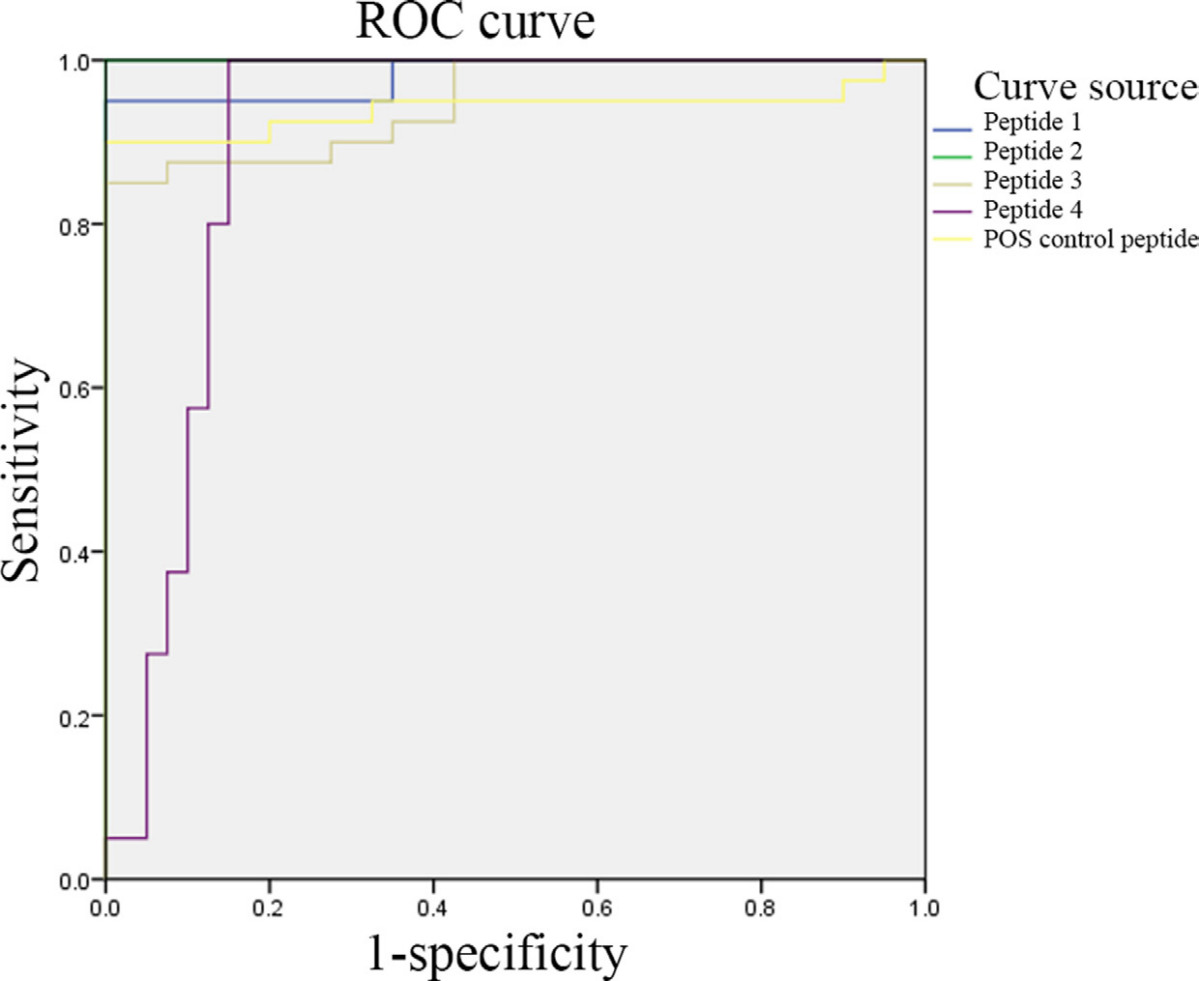
ELISA-based validation for the significantly differentially expressed peptides Peptide 1: (P0)-4; peptide 2: (P0)-11; peptide 3: DNA topoisomerase 1 (full length)-1; and peptide 4: U1-SnRNP 68/70 KDa-1.

We analyzed the results for the patient and control groups and determined the upper and lower bounds, the class intervals, and the cut-off points. We then constructed a cumulative frequency distribution table according to the selected class interval and calculated the sensitivity, specificity, and false positive rate (1-specificity) for all cut-off points. Finally, the ROC curve was plotted with sensitivity as the y axis, representing the true positive rate, and (1-specificity) on the x axis, representing the false positive rate (Figure 4).

The areas under the ROC curves for peptides 1, 2, 3, 4 and the positive control peptide were 0.983, 1.000, 0.951, 0.903, and 0.941, respectively. An ROC curve combines the sensitivity and specificity in a graphical manner, which accurately reflects the relationship between the specificity and sensitivity of an analytical method and gives a comprehensive representation of the accuracy of a test. In this test, the areas under the ROC curves were all significantly higher than 0.9; thus, the biomarkers selected in this study were of significant diagnostic value. In addition, when we measured the 75 serum samples and made comprehensive diagnoses of SLE by taking into consideration clinical symptoms, the sensitivity of the peptide array in screening epitopes of the autoantigens from SLE patients was 93.9%, and the specificity was 88.0%.

## DISCUSSION

SLE is a highly prevalent autoimmune disease that affects multiple systems and organs and is associated with a variety of autoantibodies, which makes the early stages of the disease difficult to diagnose [26–28]. All the current detection methods have common limitations, such as poor positive detection rates and low sensitivity, and antibodies can only be detected using an ELISA. Due to these issues, the detection of antibodies is not only inefficient but is also costly. Therefore, the identification of a simple, fast, and low-cost diagnostic method for SLE, with good specificity and high sensitivity, has become an urgent need. This study was designed to screen the epitopes of the autoantigens of SLE patients using peptide arrays and screen for the peptides specific to these epitopes to provide a reference for the early diagnosis and treatment of SLE.

In this study, a high-throughput peptide array was used for the first time to detect the binding epitopes of SLE-related autoantibodies. SLE-related autoantigens have been reported in many studies [29, 30], and whole autoantigens can be used to detect autoantibodies. However, the complete expression of humanized antigens is very difficult; thus, using complete antigens for high-throughput screening is impossible. In addition, the known epitopes recognized and bound by antibodies, namely, the antigenic determinants, are generally only 8–15 amino acids in length [14]. Thus, the screening of epitopes can be accomplished using peptides. Meanwhile, peptides can be generated that correspond to the smallest unit that an antibody can recognize and directly and accurately bind. This technique would achieve the goal of precise testing and could possibly be developed into a precision treatment. Since each patient produces different epitopes for autoantibody binding, we can use specific peptides to block the autoantibodies and achieve a therapeutic purpose. In this study, we first screened for the 12 antigens specific to clinical autoimmune diseases, including MD1, SMD2, SMD3, proliferating cell nuclear antigen, acidic ribosomal phosphoprotein (P)-1, P2, SnRNP-B/B’, U1-snrnp A, U1-snrnp C, U1-SnRNP 68/70 KDa, DNA topoisomerase 1 (full length), and DNA topoisomerase 1 (truncated). We then used online IEDB software to analyze and predict the epitopes of the 12 antigens. Finally, after we combined our data with data from related literature [31–36], we obtained 73 peptide indicators (Supplementary Table 1). Based on these indicators, a high-throughput peptide array was prepared. This peptide array was used to detect SLE epitopes in the serum of 50 clinically diagnosed SLE patients, with normal human serum as a positive control to further screen the SLE epitopes. The screening revealed that the expression levels of 4 indicators were significantly upregulated in all SLE samples, which indicates the potential value of their application in the diagnosis of SLE. The four indicators were acidic ribosomal phosphoprotein (P0)-4, acidic ribosomal phosphoprotein (P0)-11, DNA topoisomerase 1 (full length)-1, and U1-SnRNP 68/70 Kda. Next, we validated these 4 peptide epitopes with an ELISA and found that they are as sensitive and specific in the detection of SLE as the peptide sequence (positive control peptide) reported by others. The areas under the ROC curves all exceeded 0.9. Therefore, these 4 peptides can serve as new serum markers in the diagnosis of SLE [13].

In this study, we investigated the epitopes of the specific SLE autoantibodies using peptide arrays and validated 4 epitopes. However, since most SLE patients did not undergo autoantibody detection tests, we cannot compare these results with the conventional results of autoantibody detection using intact autoantigens. Another limitation of this study is that we only detected the epitopes of serum IgG, not epitopes bound by IgM and other antibodies. The currently reported autoantibody detection methods are all for IgG autoantibodies, and the detection of other autoantibodies is rare, which may be related to the long half-life of IgG. Seven common autoantibodies have been reported in SLE patients, and each autoantibody has varying degrees of diagnostic sensitivity and specificity. In addition, because of individual differences, the autoantibodies produced by different SLE patients are not the same, that is, each SLE patient has an individual autoantibody map. Thus, if we wish to obtain a relatively complete and reliable autoantibody map, simultaneously testing a large number of samples could screen out some of the autoantibodies with a low sensitivity and specificity [37]. Lastly, due to the strict sample enrollment conditions (first diagnosis and not receiving medical treatment within the 2 months prior to SLE diagnosis), the enrolled sample size was small. In the future, we need to increase the sample size for index verification.

In conclusion, we screened the epitopes on SLE autoantigens for the first time using peptide arrays and obtained 4 peptide indicators specific for SLE epitopes, which were validated using an ELISA. The results of this study may offer a useful reference for the early diagnosis and treatment of SLE.

## MATERIALS AND METHODS

### Patients

The patient group with active SLE comprised 50 patients from the Shaoyang Central Hospital, including 10 males and 40 females with an average age of 30.7 years. All 50 patients met the criteria described by the American College Rheumatology (ACR) for the classification of SLE [38]. The exclusion criteria included: 1) another autoimmune disease (such as rheumatoid arthritis and systemic sclerosis) or diseases that affect autophagy (such as systemic infection, cancer, and neurodegeneration) and 2) receiving medical treatment within the 2 months prior to diagnosis. Demographic data of the enrolled patients were collected and disease activity was assessed based on the SLE activity index 2000 (SLEDAI-2K) [39].

The normal control group comprised 25 healthy subjects from the People’s Hospital of Xiangyang City, Hubei Province, including 5 males and 20 females with an average age of 26.5 years. All healthy control samples were derived from the serum of healthy subjects from the hospital’s health checkup center. None of the healthy controls had a family history of autoimmune diseases, and no major disease or any infectious disease was recorded within the 3 months prior to serum collection.

This study was approved by the Ethics Committee of the Shaoyang Central Hospital, and informed consent was obtained from each participant.

### Prediction of epitopes on autoantigens using IEDB software

The IEDB is a free resource that is funded by a contract from the National Institute of Allergy and Infectious Diseases, and it offers easy searches of experimental data characterizing antibody and T cell epitopes studied in humans, non-human primates, and other animal species. Epitopes involved in infectious disease, allergy, autoimmunity, and transplants are also included. The IEDB also hosts tools to assist in the prediction and analysis of B cell and T cell epitopes. We first entered the IEDB online analysis system and selected our parameters, including “any Epitopes” for “Epitope” and “all” for “assay”. We individually entered the names of the 12 antigens related to a clinical SLE diagnosis and chose “any MHC Restriction” for “MHC Restriction”, “Humans” for “Host”, and “Autoimmune Disease” for “Disease”, followed by searching and querying. Based on the obtained peptide information for each antigen indicator and the associated literature, the search range was further narrowed, and finally, the peptide sequences associated with SLE were obtained. The 12 obtained antigens were SMD1, SMD2, SMD3, proliferating cell nuclear antigen, acidic ribosomal phosphoprotein (P)-1, P2, SnRNP-B/B’, U1-snrnp A, U1-snrnp C, U1-SnRNP 68/70 KDa, DNA topoisomerase 1 (full length), and DNA topoisomerase 1 (truncated).

### Epitope prediction and polypeptide synthesis

Linear epitope predictions of the 12 autoantigens were performed using the online Bepipred Linear Epitope Prediction software [40]. As numerous epitopes were predicted for each antigen, the eight epitopes with the highest scores for each antigen were selected for cost considerations.

The selected peptide epitopes were chemically synthesized and then purified using high-performance liquid chromatography. Molecular weight verification was performed with mass spectroscopy. A biotin-labeled random linear dodecapeptide was used as the positive control, and a random linear dodecapeptide as the negative control.

### Diagnostic peptide array for SLE

The peptide array was pretreated according to the following procedures. First, a clean slide was soaked in a 5% ammonia silane anhydrous ethanol solution for 30 min, washed with anhydrous ethanol and deionized water (3 times, 5 min each, for both), and air-dried. Next, the slide was soaked in a phosphate buffer solution (PBS) containing 2.5% glutaraldehyde for 30 min, successively washed with PBS and deionized water (3 times and 5 min each for both), and air-dried.

All peptides were synthesized by Sangon Biotech Co., Ltd., (Shanghai) and peptide indicators were prepared using a Biochip Spotting Instrument (AD3200, BioDot, USA). To prepare the peptide array, we first specified the product diagram of samples, and then according to the diagram, we compiled the sample loading protocol in the text document of the biochip sampling instrument (sampling plate, sampling position, starting and ending position of sample loading in columns, and starting and ending position of sample loading in rows). Next, we enabled the sample loading software, exhausted the pipeline, and installed the needle (400 pl/point). We ran the sampling-needle cleaning procedure and tested the water-dropping procedure. The number of slides was set at 40 per batch, the sample loading distance at 0.4 mm, and the suction volume at 10 μL. We then simulated the sample loading procedures by placing the slides onto the sample loading table and measured the height of each slide. The peptide was diluted to 0.5 mg/ml, mixed well with 1× PBS (pre-filtered with a 0.45-μm filter), and centrifuged at a low speed for 2 min. According to the established protocol, the diluted peptide was slowly and gently loaded into a 384-well plate to avoid bubbles (20 μL per well) and then centrifuged at 2000 rpm for 2 min. If loading wells were missed after the sample loading program was run, then the missed-loading program was run. Then, the instrument was turned off, and the slides were stored overnight. The slides were stored in a slide box. For long-term preservation, the slides were sealed within hermetic bags at 4°C or -20°C (with the slides exposed to room temperature for 3–4 h before the bag was opened). Note that the temperature and humidity during sample loading cannot exceed 25°C and 60%, respectively.

The procedures for the screening of the peptide arrays included blocking with PBS containing 0.1% bovine serum albumin (BSA) for 30 min. We then added serum samples and incubated the arrays for 4 h, followed by washes with PBST (PBS with Tween-20) and PBS (5 times each). Biotin-conjugated human IgG was added for a 2-h incubation at room temperature. PBST and PBS washing were performed five times, respectively. Then, a 555-streptavidin fluorescein marker was added, the samples were incubated at room temperature for 1 h, followed by washes with PBST and PBS (5 times each) and air-drying.

The scanning of the arrays was performed using Jingxin LuxScan™ 10K-B Microarray Scanners (CapitalBio Corporation, China) with a 532-nm excitation scanning wavelength (parameters: power 100%, PMT 550). Signal reading and the analysis of results were carried out using Gene Pro 6.0 software. GraphPad Prism 6 software was used to test the peptide arrays. The fluorescence of the 4 indicators, acidic ribosomal phosphoprotein (P0)-4, acidic ribosomal phosphoprotein (P0)-11, DNA topoisomerase 1 (full length)-1, and U1-SnRNP 68/70 KDa-1, in samples from SLE patients and normal controls was read and plotted using Gene Pro 6.0 software, and comparisons were made between and within groups.

### ELISA-based validation

The significantly differentially expressed peptides were diluted with a coating buffer by the addition of 100 μL into each well of the 96-well ELISA plates. After an overnight incubation at 4°C, the plates were washed with PBST and PBS (5 times each). Then, 200 μL PBS containing 0.1% BSA was added to each well. The wells were blocked at 37°C for 2 h and then washed with PBST and PBS (5 times each). Serum samples (100 μL per well) were added to the plates, incubated at 37°C for 1 h, and washed with PBST and PBS (5 times each). Then, 100 μL biotin-conjugated human IgG was added to each well, incubated at 37°C for 1 h, and washed with PBST and PBS (5 times each). Next, 100 μL horseradish peroxidase (HRP)-streptavidin fluorescein marker was added per well, incubated at 37°C for 1 h, and washed with PBST and PBS (5 times each). Freshly prepared tetramethylbenzidine (TMB) was then added to the plates (100 μL per well) as the substrate and incubated at 37°C for 15 min, followed by the addition of 50 μL 2 M concentrated sulfuric acid per well to stop the reaction, which turned the solution yellow. A microplate reader (purchased from Biotek Instruments Ltd., USA) was used to detect the absorbance of each well at 450 nm. GraphPad Prism 6 software was used to calculate the mean values of the ODs obtained from the ELISA-based validation for the 4 indicators, acidic ribosomal phosphoprotein (P0)-4, acidic ribosomal phosphoprotein (P0)-11, DNA topoisomerase 1 (full length)-1, and U1-SnRNP 68/70 KDa-1, in the samples of SLE patients and normal controls. These mean values were subtracted from the mean values for the normal controls and SLE patients. Comparisons were made between and within groups of normal subjects and SLE patients.

### Statistical analysis

Peptide array data (fluorescence signal reading) were obtained using Gene Pro 6.0 software, and the results were analyzed using GraphPad Prism 6.0 software (*t* tests). Analysis of the ROC curves and the related data were performed using SPSS software. *P* < 0.05 was considered statistically significant.

## SUPPLEMENTARY MATERIALS TABLE



## Abbreviations

SLE: systemic lupus erythematosus
ELISA: enzyme-linked immunosorbent assay
IEDB: Immune Epitope Database and Analysis Resource
