# Supplementary material for: Screening epitopes on systemic lupus erythematosus autoantigens with a peptide array
Source: Oncotarget. 2017 Sep 18;8(49):85559–67. doi: 10.18632/oncotarget.20994 (PMC5689631; doi:10.18632/oncotarget.20994)
Supplement: Supplementary file 1 [file oncotarget-08-85559-s001.pdf]

Screening epitopes on systemic lupus erythematosus autoantigens with a peptide array

SUPPLEMENTARY MATERIALS

Supplementary Table 1: Information about indicators for the SLE diagnostic peptide array

Posit-1	PO-6	P2-2	SMD3-1	SnRNP-B/B'-6	U1-Snrnpc-2	DNA topoisomerase 1 (full length)-4	U1-SnRNP68/20KDa-8	Nucleolin-3
Posit-2	PO-7	SMD1-1	SMD3-2	SnRNP-B/B'-7	U1-Snrnpc-3	DNA topoisomerase 1 (full length)-5	DNA topoisomerase 1 (truncated)-1	Nucleolin-4
Posit-3	PO-8	SMD1-2	SMD3-3	SnRNP-B/B'-8	U1-SnrnpA-1	U1-SnRNP68/20KDa-1	DNA topoisomerase 1 (truncated)-2	Nucleolin-5
buffer	PO-9	SMD1-3	SMD3-4	PCNA-1	U1-SnrnpA-2	U1-SnRNP68/20KDa-2	DNA topoisomerase 1 (truncated)-3	Nucleolin-6
PO-1	PO-10	SMD2-1	SMD3-5	PCNA-2	U1-SnrnpA-3	U1-SnRNP68/20KDa-3	DNA topoisomerase 1 (truncated)-	Nucleolin-7
PO-2	PO-11	SMD2-2	SnRNP-B/B'-1	PCNA-3	U1-SnrnpA-4	U1-SnRNP68/20KDa-4	DNA topoisomerase 1 (truncated)-5	Posit-1
PO-3	PO-12	SMD2-3	SnRNP-B/B'-3	PCNA-4	DNA topoisomerase 1 (full length)-1	U1-SnRNP68/20KDa-5	DNA topoisomerase 1 (truncated)-6	Posit-2
PO-4	P1-1	SMD2-4	SnRNP-B/B'-4	PCNA-5	DNA topoisomerase 1 (full length)-2	U1-SnRNP68/20KDa-6	Nucleolin-1	Posit-3
PO-5	P2-1	SMD2-5	SnRNP-B/B'-5	U1-Snrnpc-1	DNA topoisomerase 1 (full length)-3	U1-SnRNP68/20KDa-7	Nucleolin-2	buffer
